# Airway epithelial cells exposed to wildfire smoke extract exhibit dysregulated autophagy and barrier dysfunction consistent with COPD

**DOI:** 10.1186/s12931-018-0945-2

**Published:** 2018-11-28

**Authors:** Eugene Roscioli, Rhys Hamon, Susan E. Lester, Hubertus P. A. Jersmann, Paul N. Reynolds, Sandra Hodge

**Affiliations:** 1 Department of Thoracic Medicine, Royal Adelaide Hospital, Adelaide, South Australia Australia; 2 Deptartment of Medicine, The University of Adelaide, Adelaide, South Australia Australia; 3 Department of Rheumatology, The Queen Elizabeth Hospital, Adelaide, South Australia Australia; 4 Adelaide Health and Medical Science Building, Corner of North Terrace and George St, Adelaide, South Australia 5005 Australia

**Keywords:** Airway epithelium, Barrier function, Cigarette smoke, COPD, Environmental health, Exacerbation, Wildfire

## Abstract

**Background:**

Individuals with respiratory disease are being increasingly exposed to wildfire smoke as populations encroach further into forested regions and climate change continues to bring higher temperatures with lower rainfall. Frequent exposures have significant potential to accelerate conditions such as chronic obstructive pulmonary disease (COPD) which is characterised by an exaggerated inflammatory response to environmental stimuli. Here we employ models of human airway epithelium exposed to wildfire smoke-extract (WFSE) to examine modulation in airway epithelial cell (AEC) survival, fragility and barrier function.

**Methods:**

Submerged cultures of small airway epithelial cells (SAEC) and differentiated air-liquid interface (ALI) cultures of primary bronchial AEC (bAEC) were treated for 1–24 h with 1–10% WFSE generated from plant species found in the Australian bushland. Autophagy (LC3-II and Sequestosome), apoptosis (Poly-(ADP)-Ribose Polymerase (PARP) cleavage) and tight junction proteins were measured using western blot. Barrier function was assessed via permeability of fluorescein tracers and measuring trans-epithelial electrical resistance. The production of IL-6 was assessed using ELISA.

**Results:**

Primary epithelial models exposed to WFSE exhibited a significant blockade in autophagy as evidenced by an increase in LC3-II coupled with a concomitant elevation in Sequestosome abundance. These exposures also induced significant PARP cleavage indicative of apoptotic changes. ALI cultures of bAEC treated with 5% WFSE demonstrated barrier dysfunction with significant increases in paracellular molecular permeability and ionic conductance, and a reduction in the abundance of the tight junction proteins ZO-1 and Claudin-1. These cultures also exhibited increased IL-6 secretion consistent with the aberrant and pro-inflammatory repair response observed in the COPD airways. Further, blocks in autophagy and barrier disruption were significantly elevated in response to WFSE in comparison to similar exposures with cigarette smoke-extract.

**Conclusion:**

WFSE inhibits autophagic flux and induces barrier dysfunction in the airway epithelium. As autophagy is a central regulator of cellular repair, viability, and inflammation, targeting the block in autophagic flux may ameliorate the consequences of wildfire smoke-exposure for individuals with pre-existing respiratory conditions.

**Electronic supplementary material:**

The online version of this article (10.1186/s12931-018-0945-2) contains supplementary material, which is available to authorized users.

## Introduction

The airway inflammation and remodelling observed during chronic obstructive pulmonary disease (COPD) is driven by exogenous noxious stimuli that activate the airway epithelium, which responds by releasing alarmins and pro-inflammatory factors that promote the recruitment of inflammatory cells [1, 2]. Consequently, identifying discrete biochemical effects caused by the exacerbating factors that potentiate this chronic airway wounding scenario is a priority for COPD research [3, 4]. In addition to cigarette smoke, exposure to smoke from burning wood and the combustion of biomass for cooking and heating which is prevalent in underdeveloped countries are associated with the development of COPD [5]. Further to this, the extensive clouds of soot and gas produced during a wildfire event or prescribed burns are a far less avoidable form of smoke exposure, and have the potential to exacerbate the respiratory symptoms of the surrounding population for an extended period of time (reviewed in [6]).

There are a number of reasons why wildfire smoke is an important consideration for individuals with COPD; 1) the rising incidence of COPD, which is already the third leading cause of death in the USA [7], 2) the expansion of metropolitan populations in proximity to forested regions and prescribed burns [8], 3) the increasing frequency of wildfire events associated with elevated atmospheric temperatures and reduced precipitation [9], and 4) the pressure on hospital resources during large scale crises. Further, advanced degeneration of the airways generally effects elderly sufferers of COPD who may be unable to escape the rapid onset of wildfire smoke, and are therefore more likely to be exposed to higher levels of the particulate matter that can penetrate into the lower airways [6, 9–11]. While exposure to wildfire smoke is less frequent than, for example, exposure to biomass smoke within dwellings, wildfire smoke can cover vast populated regions with highly concentrated and volatile chemicals (e.g. acrolein, benzene, and phenols) produced after the combustion of unprocessed organic material which can persist for several hours [5, 6, 9, 10]. As a result, there is a high probability that individuals suffering from COPD will experience an exacerbation of their symptoms that requires clinical support, and during a period when hospital resources may be limited by other emergencies related to the wildfire. This has been reported in several epidemiological studies which show a statistically significant increase in COPD exacerbations as a result of exposure to wildfire smoke, and an increase in hospital admissions to manage the respiratory symptoms beyond the efficacy of self-administered medications [6, 9, 12, 13]. Indeed, in situations where people are exposed to intense heat or have a severe respiratory infection, co-exposure to prolonged periods of smoke may cause damage to the airway epithelium that promotes the pathogenesis of COPD [9]. This scenario is particularly relevant in countries such as Australia, which has a high prevalence of respiratory disease and where agricultural communities are required to conserve highly combustible adjacent bushlands that have evolved with bushfires as a normal phenomenon that promotes revegetation [8].

Dysfunction of the epithelial compartment in response to cigarette smoke exposure is known to contribute to the hall mark features of COPD, such as the protracted secretion of inflammatory factors, recruitment of harmful immune cells and production of cytokines such as TGF-β that remodel the airways (e.g. [3, 14]). Given that airway epithelium is the immunological interface for harmful environmental stimuli presented by the atmosphere, it then follows that airway epithelial cells (AEC) are major orchestrators of the nature and magnitude of the inflammatory response and exacerbations suffered by individuals with COPD that are exposed to wildfire smoke. Further to this, any deficits in mucociliary function caused by the extreme air temperatures associated with wildfire smoke will have immediate consequences for effective gas exchange at the alveoli. Surprisingly, there is a paucity of data describing how the airway epithelium responds to wildfire or any other form of environmental smoke exposures. Limited studies have reported that smoke exposures related to the combustion of wood or biomass initiate cytotoxic changes in the epithelium caused by the generation of reactive oxygen species, often due to dysregulation of the enzymes that counter the accumulation of intracellular oxygen radicals (e.g. [15–17]). One study has shown that chronic exposure to biomass smoke causes morphological changes to the epithelial compartment consistent with emphysema and the small airway damage elicited by cigarette smoke [18]. In line with this, another group has shown that the epithelium can undergo metaplastic and dysplastic changes in response to chronic exposure to the pollutants in biomass smoke [19]. However, more information is needed to further our understanding of how the smoke generated by the combustion of forested regions, which has a different composition to other forms of environmental smoke, impacts the viability and function of the immunological barrier imparted by the airway epithelium.

Central to the normal function of the airway epithelium is the maintenance of a semi-permeable barrier, appropriate immunological surveillance and reactivity to environmental stimuli, and normal viability and turnover with the underlying progenitor cells. We and others have reported that these characteristics are perturbed during COPD and in various models of cigarette smoke exposure, and is instead replaced by a leaky, fragile barrier which is composed of AEC with a low activation threshold [14, 20, 21]. We and others also showed that a disease-related block in autophagy is a fundamental phenomenon that potentiates many of the pathological alterations ascribed to the epithelium (and other airway cells) in the context of COPD, such as susceptibility to oxidative stress, accelerated senescence and apoptosis, and a pro-inflammatory phenotype [22, 23].

Hence, here for the first time we employ primary human AEC to examine the effects of wildfire smoke using an acute exposure model. We report that while AEC exposed to wildfire smoke extract (WFSE) exhibited a similar tendency for unscheduled apoptosis when compared with cells exposed to cigarette smoke extract (CSE), WFSE produced a significant increase in epithelial permeability and blockade in autophagic flux. These findings suggest that individuals who suffer from COPD have a heightened risk of exacerbations during a wildfire event which can be worsened by the increasing use of therapeutics such as macrolides that block autophagic flux.

## Methods

### Statement of ethics

Ethics approval to perform bronchial brushings to establish primary airway cultures was obtained from the Royal Adelaide Hospital Human Research Ethics Committee, and the experiments were conducted with the understanding and the written consent of each participant.

### Culture of the 16HBE14o- and small airway epithelial cells (SAEC)

The 16HBE14o- airway epithelial cell line was a generous gift from Dr. Dieter C. Gruenert (University of California, San Francisco, CA). 16HBE14o- cells were cultured in MEM/10% FCS, with 1% penicillin/gentamicin and L-glutamine (all: Life Technologies Australia Pty Ltd., VIC, Australia) in 6-well culture plates. Commercially available primary SAEC were propagated using complete SAEC growth media (both Lonza Australia Pty Ltd., VIC, Australia) on type-I rat tail collagen coated 6-well plates (Life Technologies). For both, cells were seeded into the respective 6-well plate system and were subsequently exposed to treatments at 75–80% density on the following day. Both were subject to humidified, 37 °C, and 5% CO_2_ growth conditions.

### Air-liquid interface culture model of differentiated bronchial airway epithelial cells (bAEC)

Bronchial brushing was performed by the physicians at the Royal Adelaide Hospital’s Department of Thoracic Medicine. Consenting participants reported no history of chronic respiratory disease and were never smokers (*n* = 9; FEV1/FVC 94% (±15); four females; median age 43 ± 17 years [SD]). Samples were dissociated from the brush by gentle vortex into RPMI media with 10% FCS, with 1% penicillin/gentamicin and L-glutamine, pelleted by centrifugation, resuspended into Bronchial Epithelial Growth Media (Lonza), and supplemented with 2% Ultroser G serum substitute (Pall Life Sciences, Cergy-Saint-Christophe, France). The cell suspension was passed through a 25-gauge needle five times and then transferred onto anti-CD68 (Dako, Glostrup, Denmark)-coated culture dishes for 20 min to deplete macrophages. The suspended cells were then transferred to collagen-coated T25 flasks (Sigma-Aldrich, Castle Hill, NSW, Australia) to propagate basal progenitor AEC. When the cells achieved 80–90% density (7–10 days), they were detached using primary AEC subculture reagents (Lonza), resuspended in bronchial Air-Liquid Interface (ALI) Growth Media (Lonza), and transferred to type I collagen (StemCell Technologies, Sunrise Beach, QLD, Australia)-coated Transwells (0.4 μm pores, 6.5 mm diameter; Sigma-Aldrich), at a seeding density of 9 × 10^4^ cells per well. Once cells achieved 100% density (4–6 days), the media was removed from the apical and basal reservoirs, and bronchial ALI Differentiation Media (Lonza) was added to the basal reservoir. Cultures were included in ex vivo models when mucociliary differentiation was observed (Additional file 1: Figure S1), and transepithelial electrical resistance (TEER) (EVOM2; World Precision Instruments, Sarasota, FL) exceeded 500 Ω.cm^2^ (24–28 days) to demonstrate the formation of a continuous epithelium across cultures.

### Cigarette smoke extract and wildfire smoke extract exposures

CSE was generated and applied to the AEC culture models at 10% in the respective culture medium for each cell type, as previously reported [22, 24, 25]. WFSE conditioned media was generated in a similar manner with modifications, using biomaterial from the genera *Eucalyptus* and *Acacia* which constitute the majority of Australian vegetation. Equal weights of flora species that are indigenous or common introductions to the bushfire prone region of the Adelaide Hills, South Australia were combined: *Acacia baileyana* (Cootamundra wattle) leaves and stems, *Acacia melanoxylon* (blackwood) leaves and stems, *Acacia vestita* (weeping acacia) leaves and stems, *Eucalyptus camaldulensis* (river red gum) leaves and *Eucalyptus globulus* (blue gum) leaves. Each species was blended separately using a CG2B spice grinder (Breville, NSW, Australia). The blended material was weighed and equal portions were mixed together, half of the mixture was frozen immediately at − 80 °C (‘wet’) while the other half was dehydrated using a DT5600 food dehydrator (Sunbeam, Botany, NSW, Australia; ‘dried’) for 4 h, at setting two (approximately 55 °C) then stored in a desiccator. To prepare the 100% stock WFSE, smoke from 2 g of ignited foliage (1.5 g dried plus 0.5 g wet), a mass proportionate to the cigarette mass used for 100% CSE, was bubbled through 20 mL of HEPES buffered saline solution using a vacuum pump. A mixture of dry and wet foliage was used to approximate the combustion of dead and live components of forest material, and this ratio also provided a continuous burn rate that was comparable to the research cigarettes. pH was adjusted to neutral and aliquots were stored at − 80 °C. For initial optimisation experiments, concentrations of 1–10% (in culture media) were investigated, and a maximal concentration of 5% was applied in all experiments.

### Lactate dehydrogenase necrosis assay

The measurement of lactate dehydrogenase (LDH) release into the media from compromised cells was performed according to the manufacturer’s instructions (Roche, Penzberg, Germany), to quantify necrosis as a result of the cytotoxic effects imparted by the exposures. Maximum LDH release was determined for each culture by lysing the cells in a single well with detergent. Absorbance values were analyzed using a fractional (logit link) regression model with R statistical software (release 3.2.3 [26]) and results expressed as percent cell death.

### Western blot protein analysis

Protein was isolated in situ, and western analysis performed as previously described [27]. Blots were probed using antibodies directed to Claudin-1, Occludin-1, ZO-1 (Thermo Fisher Scientific, North Ryde, Sydney, NSW, Australia), LC3, poly (ADP)-ribose polymerase (PARP), Sequestosome (Cell Signaling Technology, Boston, MA), Bcl2, NF-κβ (Santa Cruz Biotechnology, Dallas, TX) and β-actin (Sigma-Aldrich) followed by matching horseradish peroxidase-conjugated secondary antibodies (R&D Systems, MN, USA). Detection was performed using ECL Prime chemiluminescent substrate (GE Healthcare, Buckinghamshire, UK). Densitometry of histogram analyses was performed using Multi Gauge software (V3.1 Fugifilm, Tokyo, Japan). Density scores were normalized to both β-actin and the untreated control, and analyzed using a bootstrapped gamma regression model (log link) and stratified by replicate. The statistical analysis was performed using R statistical software (release 3.2.3) and results expressed as relative abundance.

### Measurement of epithelial electrical impedance

Electrical impedance (to restrict ionic conductance) imparted by differentiated epithelial cultures was measured to determine the integrity of tight junctions (TJ) as previously reported [21]. Culture plates containing transwell ALI cultures were allowed to acclimatise for 30 min on a 37 °C heating platform (Lecia Biosystems, Mt. Waverley, VIC, Australia) within a biosafety cabinet before reading electrical impedance using the EVOM2 Ohm meter (World Precision Instruments, Sarasota, FL, USA). Raw resistance values were converted to TEER values by subtracting the resistance of a blank transwell insert and factoring for the surface area of the membrane support (0.33 cm^2^). The data was analyzed using a linear regression model with standard errors adjusted for the clustering of results within participants. Marginal mean and contrast values were determined to identify the effect of the treatments relative to the control and 10% CSE exposures. The statistical analysis was performed using R statistical software (release 3.2.3), and results expressed as Ω.cm^2^.

### Assessment of epithelial paracellular permeability

Paracellular permeability was assessed in the transwell ALI culture system using a fluorescent tracer assay method as previously reported [21]. Sodium fluorescein (NaFl; Sigma Aldrich) was suspended in growth media (0.5 mg/mL) and applied to the apical reservoir after the treatment period. The concentration of NaFl that permeated across the epithelial layer and into the basal reservoir was determined using fluorimetry (FLUOstar Optima, BMG Labtech, Mornington, VIC, Australia) for each time interval. The data was analyzed using a gamma (log link) generalized linear regression model with standard errors adjusted for the clustering of results within participants. Marginal mean and contrast values were determined to identify the effect of the treatments relative to the control and 10% CSE exposures. Statistical analysis was performed using R statistical software (release 3.2.3) and results expressed as μg/mL of NaFl.

### Enzyme-linked immunosorbent assay (ELISA)

Conditioned media from bAEC ALI cultures were assessed for IL-6 secretion using ELISA following the manufacturer’s protocol (Life Technologies, Mulgrave, VIC, Australia). Linear regression of the standard curve was used to convert absorbance values into pg/mL. The statistical significance of the outcome measures was determined using a linear regression model stratified by replicate. Statistical analysis was performed using R statistical software (release 3.2.3) and results expressed as pg/mL.

## Results

### Cytotoxic responses in AEC exposed to WFSE

**Fig. 1:**
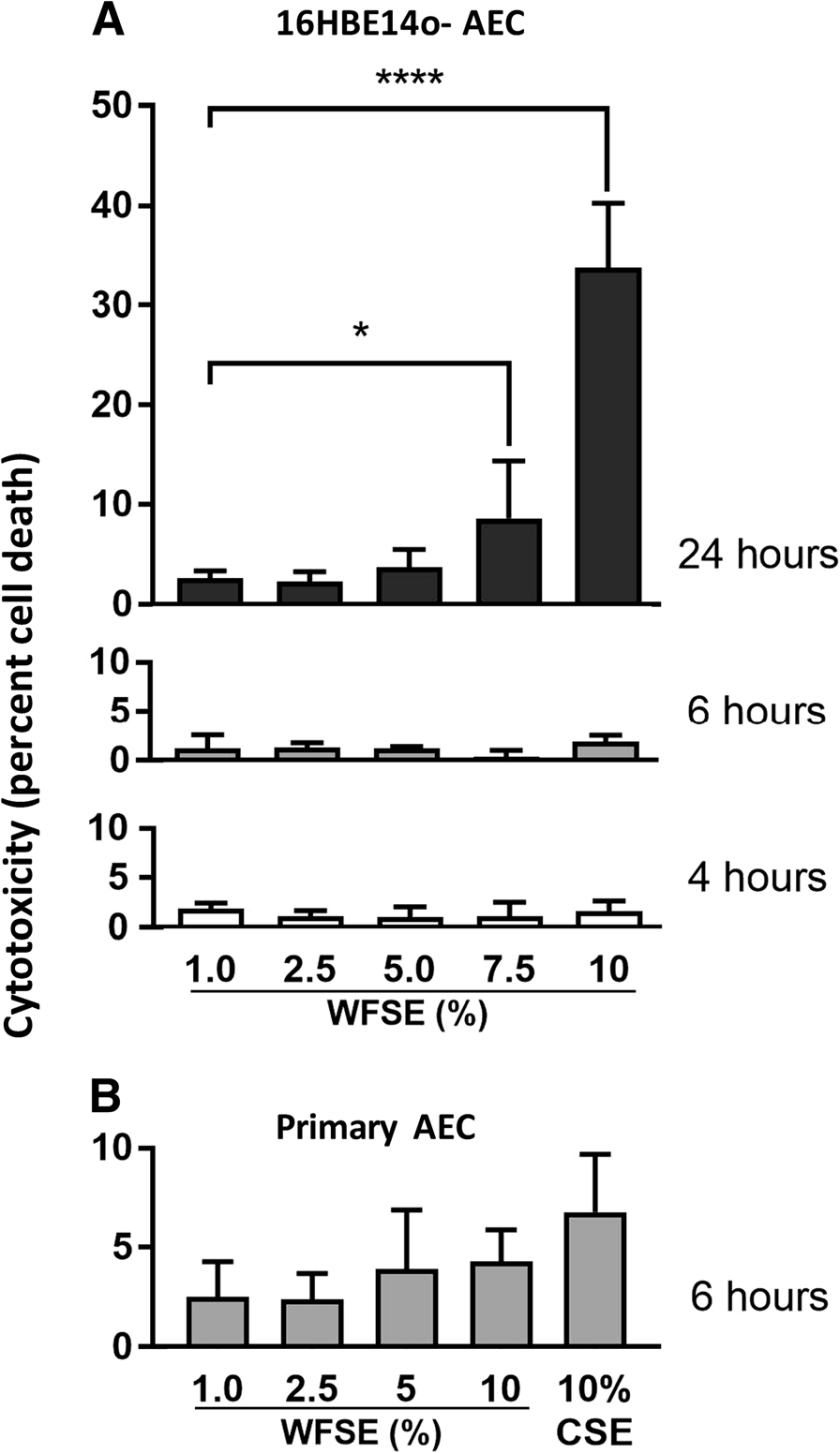
Necrosis in human airway epithelial cells exposed to WFSE. **a** Application of 7.5–10% wildfire smoke extract (WFSE) into cultures of the 16HBE14o- airway epithelial cell line elicits a significant cytotoxic response (release of lactate dehydrogenase from compromised cells) vs. control cells, after a 24 h exposure period. Over shorter periods, cytotoxicity was not significantly induced compared to base-line levels of cell turnover. **b** Primary small airway epithelial cells did not demonstrate a significant cytotoxic response (compared to the control exposure) after 6 h using a similar treatment regimen, and for the 10% cigarette smoke extract (10% CSE) exposure.Intervals are SE. * *P* ≤ 0.05, **** *P* ≤ 0.0001. Each time point represents *n* = 3 experiments

The 16HBE14o- cell line was subject to increasing exposures of WFSE to determine the treatment parameters that elicit biochemical effects without significant cytotoxicity. Significant cytotoxicity was identified after 24 h with 7.5 and 10% WFSE for 16HBE14o- cells (*P* < 0.05 and *P* < 0.0001 respectively, *n* = 3; Fig. 1a). An exposure period of 6 h was therefore chosen to examine whether the primary SAEC cultures were similarly resistant to the cytotoxic effects of WFSE for this treatment regimen, and in comparison to 10% CSE. The 6 h exposure period did not cause significant cellular necrosis (vs. the control) for each treatment (joint *P*-value over the treatment regimen = 0.59, *n* = 3), and the proportion of cell death was similar for 10% WFSE and 10% CSE exposures (4.3%, *P* = 0.51 and 6.8%, *P* = 0.23 cytotoxicity respectively; Fig. 1b). Given 6 h is also consistent with the time period an individual may be exposed to the atmospheric toxins produced by a forest fire event (e.g. to protect their property or to escape the smoke), this exposure period was used in subsequent experiments.

### WFSE potentiates apoptosis and a block in autophagic flux in SAEC

**Fig. 2:**
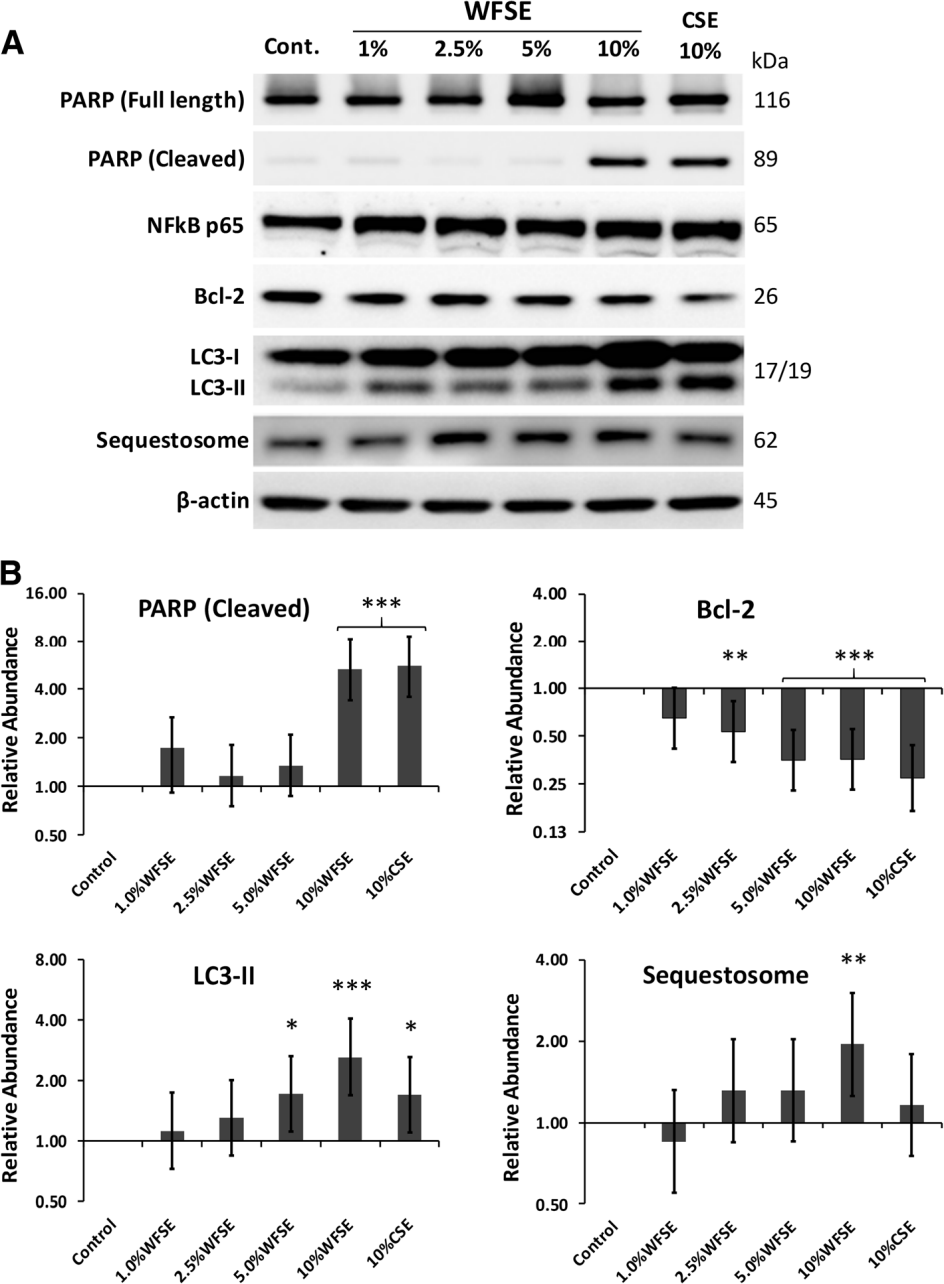
Inhibition of autophagic flux and apoptotic changes in primary SAEC exposed to WFSE. **a** Small airway epithelial cells subject to 10% wildfire smoke extract (WFSE) for 6 h undergo apoptotic changes as evidenced by the cleavage of the caspase-3 substrate Poly (ADP-ribose) polymerase (PARP). This may be due to the decrease in the inhibitor of the extrinsic apoptotic pathway Bcl2, and a blockade in normal autophagic flux as determined by the increase in the essential autophagy protein Microtubule-Associated Protein 1A/1B-Light Chain-3-II (LC3-II; lower band), simultaneous with increased Sequestosome, which is degraded with the cargo it shuttles to the autophagosome. While 10% cigarette smoke extract (CSE) also initiated an apoptotic response concomitant with a reduction in Bcl2, this was not accompanied by a block in autophagic flux, as Sequestosome abundance remained relatively low in relation to the elevation in LC3-II. The expression of the pro-inflammatory transcription factor NF-κβ subunit (p65) did not significantly increase in this model. **b** Histogram analyses of protein expression density scores. Protein expression was baselined to the abundance in the untreated sample, and normalized to the expression of β-actin. Intervals are 95% CI, and significance compared to the control sample can be identified when confidence intervals do not intersect 1 for the Y-axis. *, *P* ≤ 0.05; **, *P* ≤ 0.01 ***, *P* ≤ 0.001 for *n* = 3 experiments

SAEC cultures were exposed to WFSE for 6 h and examined via western blot analysis for programmed cell death. While all cultures were indistinguishable from the control cells on routine microscopic examination, significant apoptosis was measured via the cleavage of the caspase-3 substrate PARP in cells exposed 10% WFSE (5.32-fold increase vs. the control, CI [1.90, 2.94], *P* < 0.001; Fig. 2a, b). A similar frequency of PARP cleavage was observed for the 10% CSE exposure (5.56-fold increase, CI [1.98, 3.07], *P* < 0.001), suggesting a similar component contained in either WFSE and CSE may potentiate programmed cell death. Indeed, Bcl2, a potent inhibitor of mitochondrial depolarisation was significantly downregulated by the 2.5–10% concentration range, with a similar reduction for both 5 and 10% WFSE (− 2.83-fold, *P* < 0.001, and − 2.81-fold, *P* < 0.001, respectively; refer to Fig. 2). However, the reduction in Bcl2 was significant for each of these treatments (including the 10% CSE exposure), this finding alone could not explain why the lower concentrations of WFSE did not elicit apoptosis in the manner that was observed for the 10% WFSE treatment.

The autophagic cell survival process was examined for evidence of dysfunction to elucidate why 10% WFSE promoted apoptosis in SAEC. The abundance of LC3-II (the lipidated form of LC3 that is an essential component of the autophagosome) was measured with the adapter protein Sequestosome which is co-degraded with cargo it shuttles to the autophagic apparatus, to identify alterations in autophagic flux. The 10% WFSE exposure promoted a relatively large increase in LC3-II (2.62-fold, CI [1.43, 1.69], *P* < 0.001), and a concomitant increase in Sequestosome (1.92-fold, CI [0.69, 1.06], *P* < 0.01), which indicates a significant block in the autophagic apparatus to cope with an increase in (for example) damaged organelles and misfolded proteins (Fig. 2). The increase in LC3-II production was also significant for the 5% WFSE and 10% CSE exposures (1.71-fold, CI [0.60, 0.94], *P* < 0.05, and 1.70-fold, CI [0.60, 0.93], *P* < 0.05 respectively), but without a significant concomitant increase in Sequestosome. This was also observed in cultures of phorbol myristate acetate differentiated THP-1 macrophages exposed to the 10% WFSE treatment (Additional file 2: Figure S2), suggesting that a common defect in autophagic flux is elicited by 10% WFSE (vs. the 10% CSE exposure) in SAEC and THP-1 macrophages. Further, while autophagy is also an influential inhibitor of inflammation, the abundance of the p65 NF-κβ protein (a pro-inflammatory transcription factor) was as not significantly upregulated in this model (Fig. 2).

### Differentiated bAEC exposed to WFSE exhibit a fragile and leaky barrier phenotype

Barrier function was examined to determine whether WFSE exposure produces a fragile epithelial phenotype in primary bAEC differentiated at an ALI. TEER was quantified to assess the integrity of the TJ complexes by quantifying their ability to impede an electric current applied across the epithelial layer (i.e. ionic conductance of the paracellular pathway). The tendency for smoke extract to elicit a reduction in TEER (an increase in ionic conductance) vs. the control exposure is observed at 6 h for the 2.5–10% WFSE and 10% CSE treatments (*P* < 0.001 for each, *n* = 3; Fig. 4a). The dose dependent reduction in TEER (vs. the control) continues up to 24 h for these treatments (contrast *P* value < 0.001 for each over the exposure period), and extends down to 1% WFSE (contrast *P* value = 0.001; Additional file 3: Table S1). However, only 10% WFSE produces a significant overall reduction in bAEC TEER in comparison to the 10% CSE exposure (*P* = 0.001 over the exposure period).

**Fig. 3:**
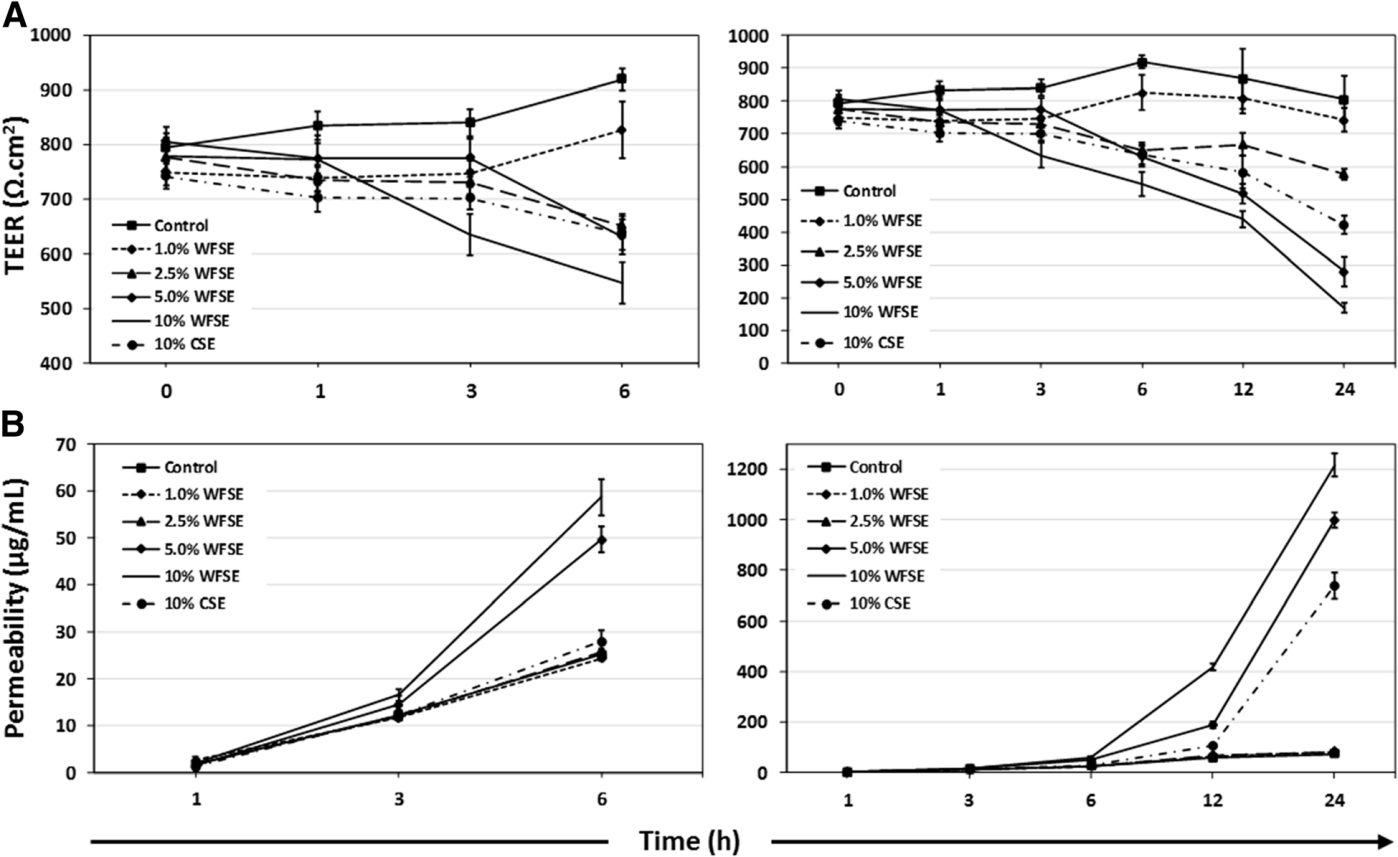
Differentiated primary human bAEC exposed to WFSE exhibit a fragile epithelial phenotype Trans-epithelial electrical resistance (TEER; ionic conductance of the paracellular pathway) and passage of sodium fluorescein tracers (paracellular molecular flow) were quantified for primary bronchial airway epithelial cells that were differentiated in a transwell culture system. **a** A reduction in electrical impedance (vs. the control exposure) is evident for the 2.5–10% wildfire smoke extract (WFSE) treatments after the 6 h exposure interval (left graphic). This relationship is maintained after 24 h (right graphic) when the maximum reduction in electrical resistance is observed for 5–10% WFSE, and the effect of 10% cigarette smoke extract (10% CSE) exceeds 2.5% WFSE. **b** The passage of sodium fluorescein tracers from the apical to the basal reservoir is evident for the 5 and 10% WFSE exposures at the 6 h interval (left graphic). At the 24 h interval the influence of 5–10% WFSE potentiates a marked increase in paracellular molecular flow, and are followed by 10% CSE treatment. Data is representative of n = 3 cultures from different individuals. Intervals are SE. For clarity “*” were omitted from these graphics, and *P* values are reported in Additional file 3: Table S1

Perhaps a more informative analysis of TJ integrity in the context of exposure to the fine particles contained in wildfire smoke and COPD, is the assessment of paracellular molecular flow. Hence, we next assessed the passage of fluorescent tracers between bAEC to quantify changes in permeability of the paracellular pathway. Unlike the observations for TEER, the passage of NaFl tracer is most noticeably increased after 6 h exposure for only the 5 and 10% WFSE treatments (*P* values < 0.001, *n* = 3), while the other treatments cluster with the control exposure (Fig. 3 A; note that maintaining tracer dye even on control cells effects a small increase in epithelial permeability). Further, the 5 and 10% WFSE exposures potentiate a marked increase in paracellular permeability beyond 6 h, to the 24 h assay period, and which are significantly more influential than the effect imparted by 10% CSE (contrast P value < 0.001 for 5 and 10% WFSE exposures, vs. 10% CSE; Additional file 3: Table S1). While the effect of necrosis and apoptosis would contribute to the increase in permeability for the 10% WFSE and 10% CSE exposures after 6 h (Figs. 1 and 2), the conditions imparted by 5% WFSE may therefore be largely confined to the disruption of TJ complexes. Hence, next we asked whether the expression of the proteins that constitute the TJ apparatus is down-regulated in bAEC exposed to 5% WFSE.

### WFSE potentiates a downregulation of tight junction proteins in differentiated bAEC

**Fig. 4:**
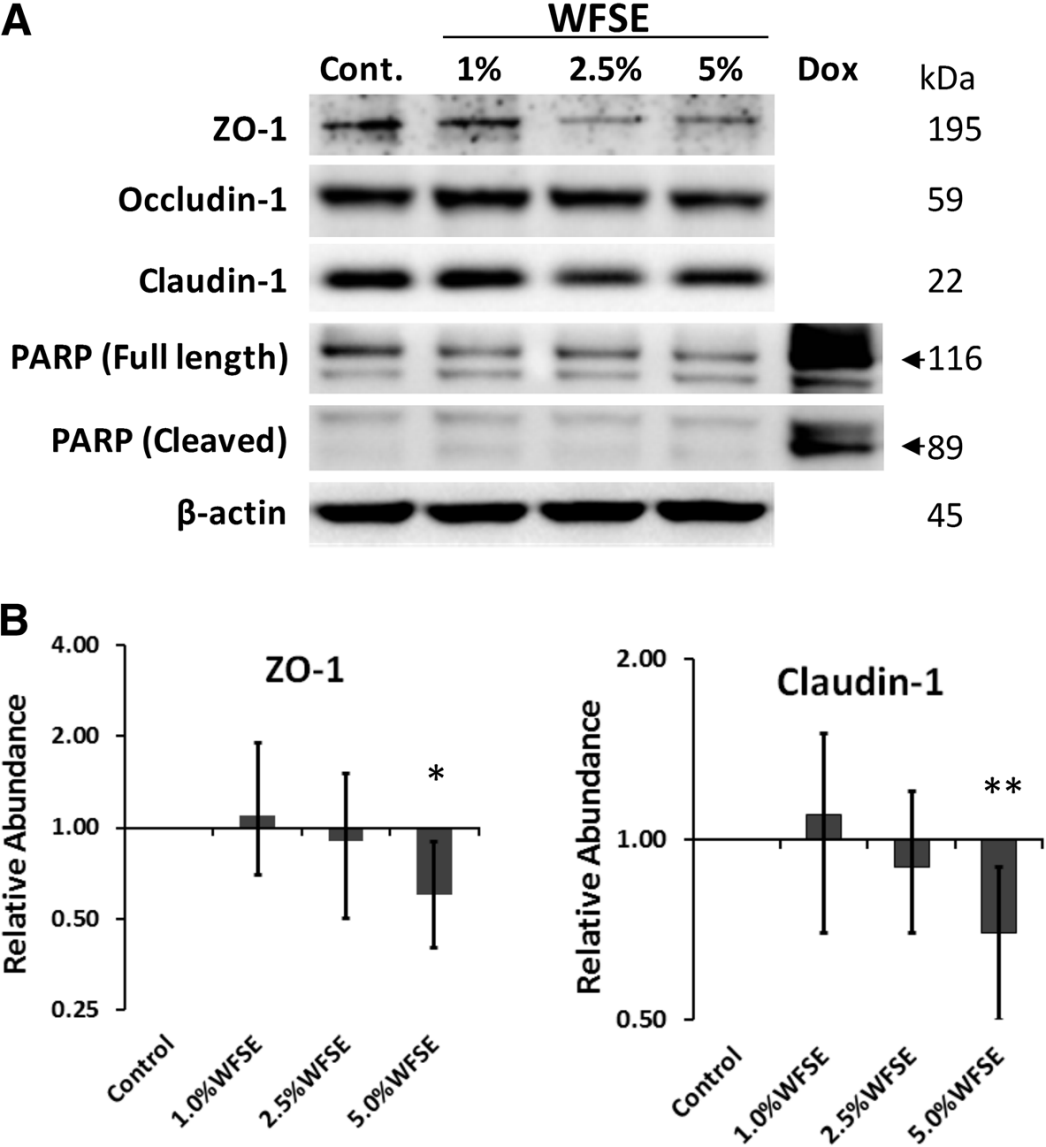
Differentiated primary human bAEC exposed to WFSE exhibit a reduction in TJ proteins. **a** Western analysis of tight junction proteins from primary human airway epithelial cells grown at an air-liquid interface showing a reduction in ZO-1 and Claudin-1 for the 5% wildfire smoke extract (WFSE) exposure after 6 h. While the abundance of Occludin-1 followed a similar trend for 5% WFSE exposure, its downregulation did not reach significance in this model. Apoptosis as evidenced by the cleavage of the caspase-3 substrate Poly (ADP-ribose) polymerase (PARP) was not detected. As significant apoptosis was not anticipated, a doxorubicin exposure (1 μM) was included to produce a caspase-mediated apoptotic outcome as a positive control to identify the products of caspase-3-mediated PARP cleavage in the western blot. **b** Histogram analyses of protein expression density scores. Protein expression was baselined to the abundance in the untreated sample, and normalized to the expression of β-actin. Intervals are 95% CI, and significance compared to the control sample can be identified when confidence intervals do not intersect 1 for the Y-axis. *, *P* ≤ 0.05; **, *P* ≤ 0.01 for *n* = 3 experiments using cells derived from different participants

The proteins that make up the TJ apparatus are primary factors responsible for maintaining a selectively permeable barrier that is established between neighbouring epithelial cells. The exposure range of WFSE was confined to 1–5% WFSE as apoptosis was not detected for these concentrations after 6 h in SAEC (Fig. 2). The essential TJ proteins ZO-1 (connects Claudin and Occludin to the actin cytoskeleton) and Claudin-1 (links adjacent cells through another Claudin-1 protein) are significantly downregulated by 5% WFSE exposure in differentiated bAEC (− 1.70-fold, *P* < 0.05, 95% CI [− 1.90, − 1.11], and − 1.43-fold, *P* < 0.01, 95% CI [− 1.30, − 1.11], respectively; *n* = 3; Fig. 4a, b). Occludin-1 (links adjacent cells through another Occludin-1 molecule) demonstrates a similar pattern of downregulation with increasing concentrations of WFSE exposure (Fig. 4a), but this was not significant for 5% WFSE in the context of this model (*P* = 0.10). Minimal detectable cleavage of PARP (vs. a doxorubicin positive control) indicates that cell death was not a significant influencing factor that contributed to a reduction in the abundance of TJ proteins (Fig. 4a).

### Differentiated bAEC exposed to WFSE secrete elevated levels of IL-6

**Fig. 5:**
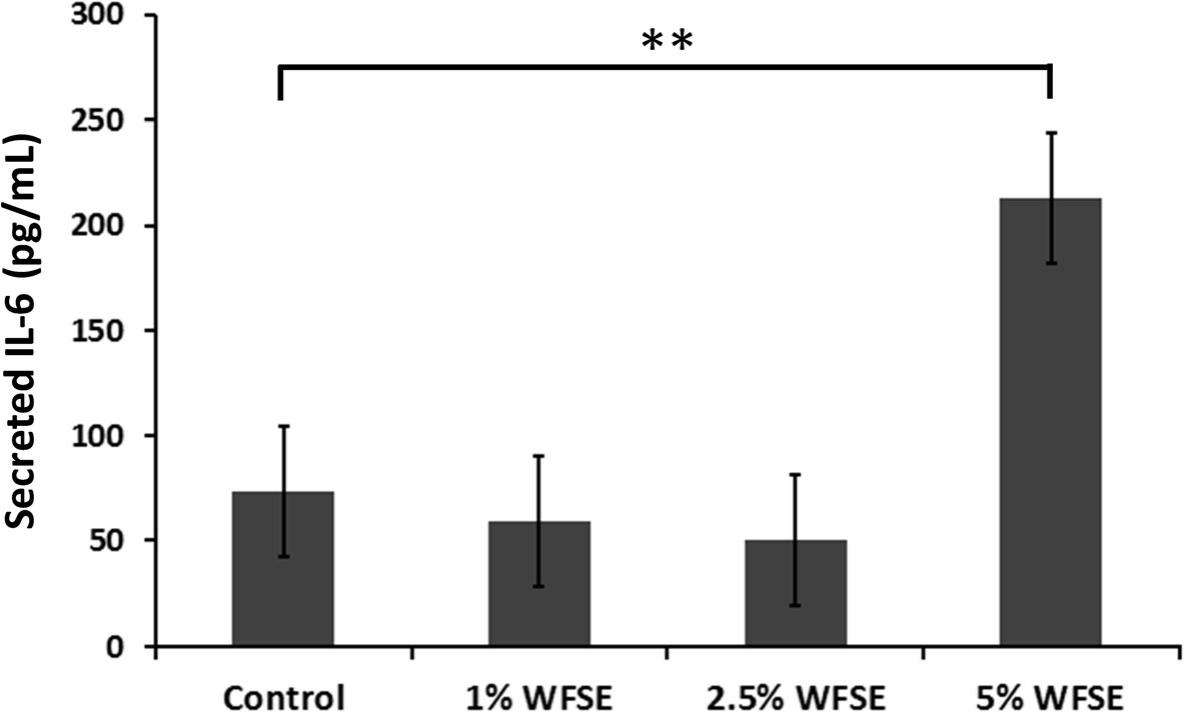
Differentiated primary human bAEC secrete IL-6 in response to WFSE. Differentiated air-liquid interface cultures of primary human bronchial airway epithelial cells were exposed to increasing concentrations of wildfire smoke extract (WFSE) for 6 h. The secretion of IL-6 was significantly increased from bAEC treated with 5% WFSE vs. the control exposure. Intervals are standard error. **, *P* ≤ 0.01 for *n* = 3 experiments using cells derived from different participants

To determine whether WFSE produces a pro-inflammatory phenotype, the secretion of IL-6 was quantified in the conditioned media obtained from the bAEC ALI cultures that were used to examine the expression of TJ proteins. While measures of IL-6 fluctuated between the 1–2.5% WFSE treatments at 6 h, a significant induction of IL-6 secretion was detected for the bAEC cultures subject to 5% WFSE (contrast regression mean vs. control = 140 pg/mL ± 33[SE], *P* = 0.005; joint *P* value over all treatments = 0.003; *n* = 3; Fig. 5). As IL-6 also promotes epithelial repair, this finding is consistent with a pro-inflammatory repair response brought about by the injurious extracellular stimuli elicited by exposure to 5% WFSE.

## Discussion

There is a paucity of information describing the influence of wildfire smoke exposure as a contributing factor in respiratory disease. Effective function of the respiratory epithelium is a central requirement for the airways to counter the immediate challenges presented by the airborne particles and gases produced during a forest fire. This is an even more important consideration when the airway epithelium is dysfunctional, as is the case for COPD, where it exhibits a complex wound healing phenotype. Here, for the first time, we applied WFSE to primary models of human AEC in the context of a moderate exposure period (6 h) to approximate the situation AEC may encounter during exposure to wildfire smoke (e.g. farmers defending a property). We found that while treatment with either WFSE or CSE produces similar cytotoxic and programmed death responses, exposure to WFSE elicited autophagic insufficiency and a significantly greater magnitude of barrier dysfunction in the primary human epithelial cultures.

We first asked whether epithelial cell survival was affected by WFSE, as per the situation during COPD where aberrant turnover of AEC is a frequently observed phenomenon elicited by the cigarette smoke and inflammation [28, 29]. WFSE induced a significant cytotoxic response in the normally resilient 16HBE14o- cell line (Fig. 1a). This was reduced to background levels of cell necrosis after limiting the exposure period from 24 h to 6 h for the 16HBE14o- cells and in the primary SAEC models (Fig. 1b). However, a significant apoptotic response was observed for the SAEC after 6 h exposure to 10% WFSE, as evidenced by the cleavage of the caspae-3 substrate PARP (Fig. 2). These results were consistent with a report by Pavagadhi et al. (2013) who identified significant apoptosis due to the factors generated during the combustion of forest biomass materials, albeit in the A549 alveolar cancer cell line [17]. The shift from a necrotic response to programmed cell death is in keeping with the normal (if unscheduled) clearance of damaged AEC that enables their efficient replacement by the underlying progenitor stem cells [20, 30]. This apoptotic response was accompanied by a significant reduction in the potent inhibitor of mitochondrial depolarisation, Bcl2, which protects against extrinsic apoptotic stimuli such the toxins and oxidants contained in wildfire smoke. Of note, the induction of apoptosis and the corresponding decrease in Bcl2 was similar for WFSE and CSE. Hence, an important follow-up inquiry would be to co-treat AEC with both WFSE and CSE to model the exposure of a current smoker to wildfire smoke, and examine the interaction between the signalling pathways that promote cellular survival and apoptosis in the context of COPD. Further to this, given that the treatments between 1 and 5% WFSE did not promote apoptosis suggests that the 10% WFSE exposure may overcome or otherwise inhibit the survival mechanisms that protect against programmed cell death in AEC.

Bcl2 is also an inhibitor of autophagy [31], which is a fundamental cell survival process that is central in maintaining cellular homeostasis during periods of cell stress and starvation, and its dysregulation has been consistently associated with the pathogenesis of COPD (e.g. [32, 33]). We observed a significant increase in LC3-II (an essential component of the autophagic apparatus) following 5–10% WFSE and 10% CSE treatment (Fig. 2a), which was likely a homeostatic response to the accumulation of damaged organelles and misfolded proteins that are generated by (for example) the oxidants and toxins that are present in these exposures [22, 32, 33]. Of note, and in contrast to the other exposures, we observed a significant increase in Sequestosome following 10% WFSE treatment, concomitant with the elevation in LC3-II abundance (Fig. 2a, b). As Sequestosome (an adapter protein) is normally co-degraded with the cargo it shuttles to the autophagosome, our findings indicate a block in autophagic flux beyond the homeostatic influence imparted by the induction of LC3-II. Indeed, a block in autophagic flux was also exclusively observed for the 10% WFSE exposure in THP1 macrophages (Additional file 2: Figure S2). Hence, determining whether the autophagic insufficiency is a response related to an excess of accumulating cargo or occurred due to a defect in the regulators of autophagy may be an important consideration for COPD in the context of wildfire smoke exposure. This may be particularly critical for COPD patients that are prescribed macrolide antibiotics which are potent inhibitors of autophagic flux (e.g. [34]). The consequences for AEC with reduced autophagic potential is a diminished capacity to recycle potentially harmful cytosolic debris generated by the wildfire smoke. In addition, autophagy is an influential cytoprotective process that counters oxidative stress, inflammation, and senescence which are all central aspects in the pathogenesis of COPD [32]. Further, defective autophagy is also associated with a poorly understood link with aberrant apoptosis (“autophagic cell death” [35, 36]). We have found that unto itself CSE exposure is not enough to initiate defects in autophagy in primary models of human airway epithelium without further COPD-related stimuli such as nutrient deprivation [21]. Hence, given WFSE elicited a block in autophagic flux suggests this form of environmental exposure presents a toxin (or group of toxins) that prevents effective autophagy beyond the influence imparted by a similar level of cigarette smoke.

Another disease-related phenomenon in COPD is the diminished efficacy of the junctional apparatus that maintains epithelial continuity, and which perpetuates the chronic would-repair process and airway remodelling [37]. Disruption of the sophisticated communication mechanisms that integrate adjacent AEC and progenitor cell populations (e.g. tight junctions, adherens junctions, and gap junctions) has serious consequences for the array of immunological activities that are involved in clearing noxious particles contained in environmental smoke. To examine this for WFSE, we employed an ex vivo model which closely approximates the epithelium observed in vivo (Additional file 1: Figure S1). An increase in ionic conductance of the paracellular pathway became evident after six hours for the WFSE exposures ranging from 2.5–10% (Fig. 3a). The 10% WFSE treatment elicited a significantly greater reduction in electrical impedance than 10% CSE, and this relationship became more evident beyond six hours when the effects of apoptosis and necrosis contribute to a reduction in epithelial integrity. In perhaps a more physiologically relevant analysis using fluorescent tracers (i.e. which mimics a situation whereby smoke particles passage the epithelial layer), an increase in paracellular molecular permeability was readily evident after exposure to 5 and 10% WFSE at six hours, while the effect of 10% CSE was comparable to the control (Fig. 3b). After six hours there was a vast increase in permeability for 5–10% WFSE, that was significantly greater than the effect of 10% CSE (Additional file 3: Table S1). Also, while the apoptosis and necrosis induced by 10% WFSE undoubtedly contributed to the reduction in epithelial integrity (Figs. 1 and 2), six hours treatment with 5% WFSE elicited a biochemical effect (prior to significant anoikis) that destabilised the TJ junction apparatus. In support of this, we observed a reduction in the abundance of the TJ proteins ZO-1 and Claudin-1 following six hours exposure to 5% WFSE, and in the absence of programmed cell death (Fig. 4a, b). Furthermore, while we did not detect increased activation of the potent pro-inflammatory transcription factor NFκB p65 (Fig. 2a), we observed a significant increase in IL-6 in response to WFSE (Fig. 5). IL-6 is an influential component of the inflammation in COPD, which is also secreted from AEC in response to the toxins contained in environmental smoke [38, 39]. However, in the normal situation IL-6 also plays an important role in the regeneration of AEC and co-operates with the inflammatory cells to repair the epithelial layer [40, 41]. Hence, in a scenario where wildfire smoke potentiates epithelial fragility, the secretion of IL-6 in response to defects in TJ integrity may be an important contributing factor in the chronic wound healing phenotype that is frequently ascribed to the epithelium during COPD.

Hence, we show that factors presented by wildfire smoke contribute to the deterioration of the airway epithelium, to a greater degree than cigarette smoke, most notably by blocking the fundamental cell survival processes governed by autophagy and disruption of epithelial barrier activity. To our knowledge, this is the first investigation that describes the effects of wildfire smoke using models of the epithelium in the context of COPD or any other respiratory disease. One limitation of our model is the use of the genera *Eucalyptus* and *Acacia* as a surrogate biomaterial. While these genera constitute the majority of Australia’s flora, it may be important to compare the effects elicited by wildfire products generated in countries with distinctly different vegetation. Indeed, a “standard blend” of forest-related biomass could serve as an important reference exposure in independent studies. Further, while we have progressed this inquiry beyond secondary cell lines, another important question is how AEC derived from smokers and COPD sufferers respond in similar models. Indeed, a greater level of utility can be achieved by applying an aerosol exposure system to ex vivo models to mitigate the issues related to dissolving smoke into a liquid vehicle [5], and which may be used to control particle size, gas composition, and exposure durations that vary with the distance wildfire smoke travels through the atmosphere [42]. In conjunction with a mouse model of emphysema or COPD, an aerosol system can also provide information relating to exacerbations, inflammatory responses and the consequences of smoke particle deposition in the airways, all of which potentiate the epithelial remodelling that is a hallmark feature of COPD. Given the increasing incidents of wildfires in proximity to an ever aging population who carry a high burden of respiratory disease, such inquires have significant potential to inform therapeutic and clinical strategies to ease the burden of hospitalisation caused by large scale wildfire events.

## Conclusion

Wildfire smoke has a significant negative impact on the survival of AEC and the maintenance of the immunological barrier imparted by the airway epithelium. Perhaps central to these is the disruption to the fundamental and multifaceted survival processes governed by autophagy, which are already dysregulated in COPD. Hence, targeting deficits in autophagic activity may prevent or resolve the toxic influences presented by wildfire smoke for individuals who are susceptible to exacerbations of COPD.

## Additional files

Additional file 1: **Figure S1.** Differentiated primary human bAEC cultures closely approximate the airway epithelium in vivo. An important requisite for assessing the epithelial barrier is establishing a model that closely approximates the epithelium in the human airways. **A**. Transmission electron microscopy of the apical margin of a bronchial airway epithelial cell (bAEC) grown at an air-liquid interface (ALI) exhibits the features of ciliated bAEC found in the human airway, with columnar morphology, cilia (C) containing microtubules (MT; a diagnostic feature of cilium), and the smaller villi (V) projecting from the apical membrane. Note that the mucus blanket produced by goblet cells is also present but is lost during sample preparation. **B**. Scanning electron microscopy of a fully differentiated epithelial culture showing cilia and the smaller plasma membrane villi projection. In this example, the epithelial layer supports primary human T cells that were co-cultured on the epithelium for 24 h. Not shown is high speed live cell imaging of cilia beating at a frequency of 10 Hz, and the directional movement of activated charcoal suspended in media on the epithelial layer, which is indicative of synchronous cell-to-cell co-ordination via communication through gap junction complexes. **C**. Confocal immunofluorescence analysis of a primary human differentiated bAEC culture shows an extensive network of apicolateral tight junction complexes that maintain the selectively permeable epithelial barrier. Here staining is shown for the essential tight junction protein ZO-1. Underlying nuclei are resolved (albeit out of focus to favour the resolution of the ZO-1 fluorescent signal) using the nucleic acid stain DAPI (4′,6-diamidino-2-phenylindole). **D**. A SEM micrograph of AEC grown at an ALI before the production of cilia (six days post air exposure), showing the defined apicolateral margins between adjacent cells formed by tight junction complexes (three shown with white arrows). In this example the epithelial layer supports primary human alveolar macrophages (Alv Mac) that were co-cultured on the epithelium for 24 h. (TIF 7742 kb)

Additional file 2: **Figure S2.** THP1 macrophages demonstrate a block in autophagic flux when exposed to WFSE. **A**. THP1 macrophages (differentiated using 45 nM phorbol myristate acetate for three days) were exposed to wildfire smoke extract (WFSE) for 24 h and examined for modulation in autophagy via western blot analysis. As observed in the small airway epithelial cell model, the 10% WFSE exposure potentiates a block in autophagic flux in THP1 macrophages as evidenced by an increase in the essential autophagy protein Microtubule-Associated Protein 1A/1B-Light Chain-3-II (LC3-II; lower band), simultaneous with increased Sequestosome, which is normally degraded by the autolysosome. **B**. Histogram analyses of protein expression density scores. Protein expression was baselined to the abundance in the untreated sample, and normalized to the expression of β-actin. Intervals are 95% CI, and significance compared to the control sample can be identified when confidence intervals do not intersect 1 for the Y-axis. *, *P* ≤ 0.05; **, *P* ≤ 0.01 for *n* = 3 experiments. (TIF 633 kb)

Additional file 3: **Table S1.** The main effects for bAEC barrier dysfunction elicited by WFSE.Differentiated primary bronchial airway epithelial cells were examined for barrier dysfunction by quantifying trans-epithelial electrical resistance (ionic conductance of the paracellular pathway) and permeability of fluorescent NaFl tracers (molecular flow of the paracellular pathway) applied across the epithelial layer in a transwell culture system. Results are presented for the individual exposures across the assay period intervals (i.e. incorporating each time interval from 0 to 24 h) to identify the main effects for each treatment (*n* = 3). Data for each treatment is presented relative to the control and 10% cigarette smoke extract (10% CSE) exposures. (DOCX 19 kb)

## Abbreviations

AEC: Airway epithelial cell
ALI: Air-liquid interface
bAEC: Bronchial airway epithelial cell
CSE: Cigarette smoke extract
LC3: Microtubule-Associated Protein 1A/1B-Light Chain-3
LDH: Lactate dehydrogenase
NaFl: Sodium fluorescein
PARP: Poly (ADP)-ribose polymerase
SAEC: Small airway epithelial cells
TEER: Trans-epithelial electrical resistance
TJ: Tight junction
WFSE: Wildfire smoke extract
