# Supplementary figures and images for: Airway epithelial cells exposed to wildfire smoke extract exhibit dysregulated autophagy and barrier dysfunction consistent with COPD
Source: Respir Res. 2018 Nov 28;19:234. doi: 10.1186/s12931-018-0945-2 (PMC6263553; doi:10.1186/s12931-018-0945-2)

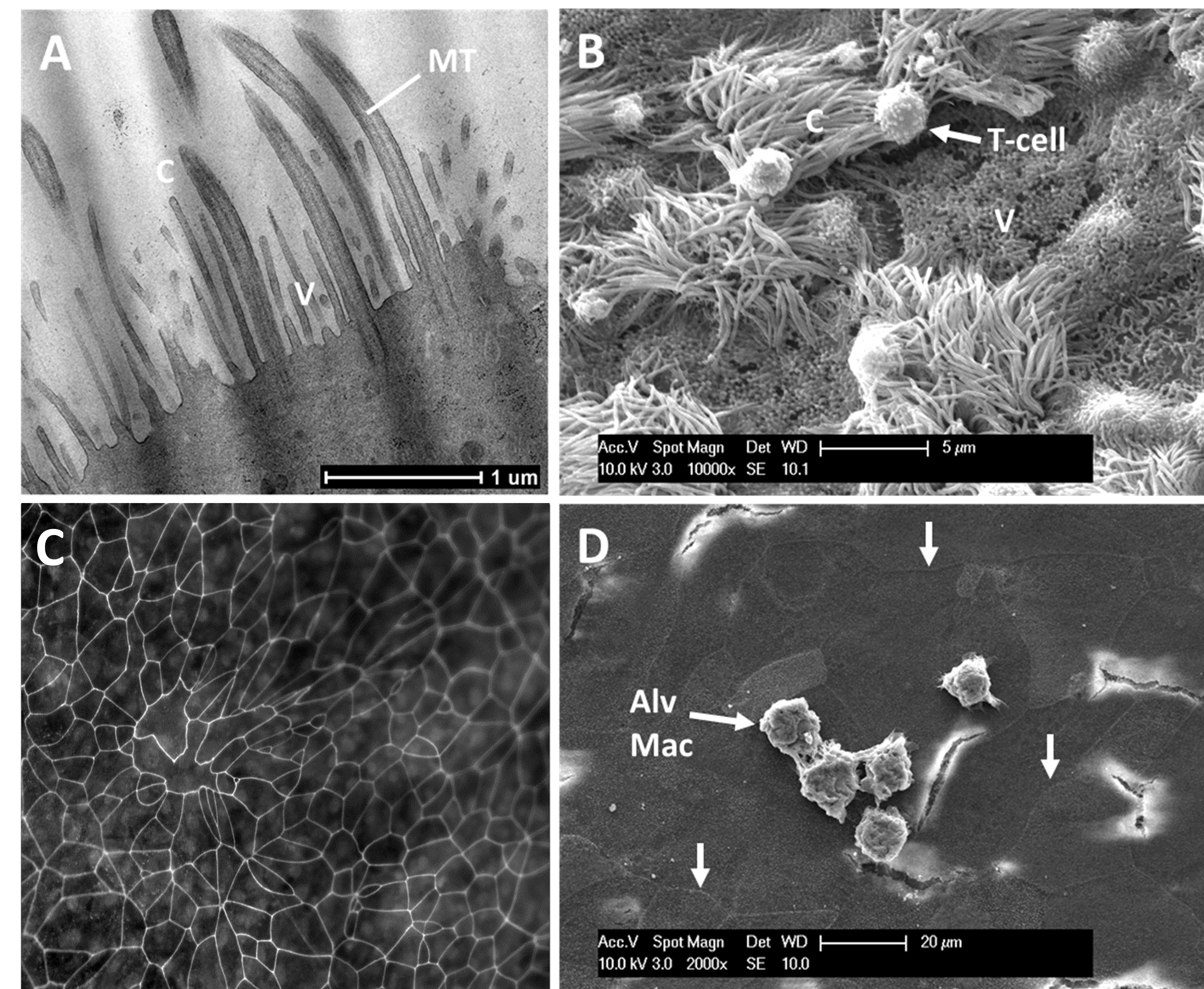

Supplement: Supplementary file 1 — Figure S1. Differentiated primary human bAEC cultures closely approximate the airway epithelium in vivo. An important requisite for assessing the epithelial barrier is establishing a model that closely approximates the epithelium in the human airways. A. Transmission electron microscopy of the apical margin of a bronchial airway epithelial cell (bAEC) grown at an air-liquid interface (ALI) exhibits the features of ciliated bAEC found in the human airway, with columnar morphology, cilia (C) containing microtubules (MT; a diagnostic feature of cilium), and the smaller villi (V) projecting from the apical membrane. Note that the mucus blanket produced by goblet cells is also present but is lost during sample preparation. B. Scanning electron microscopy of a fully differentiated epithelial culture showing cilia and the smaller plasma membrane villi projection. In this example, the epithelial layer supports primary human T cells that were co-cultured on the epithelium for 24 h. Not shown is high speed live cell imaging of cilia beating at a frequency of 10 Hz, and the directional movement of activated charcoal suspended in media on the epithelial layer, which is indicative of synchronous cell-to-cell co-ordination via communication through gap junction complexes. C. Confocal immunofluorescence analysis of a primary human differentiated bAEC culture shows an extensive network of apicolateral tight junction complexes that maintain the selectively permeable epithelial barrier. Here staining is shown for the essential tight junction protein ZO-1. Underlying nuclei are resolved (albeit out of focus to favour the resolution of the ZO-1 fluorescent signal) using the nucleic acid stain DAPI (4′,6-diamidino-2-phenylindole). D. A SEM micrograph of AEC grown at an ALI before the production of cilia (six days post air exposure), showing the defined apicolateral margins between adjacent cells formed by tight junction complexes (three shown with white arrows). In this example the epit [file 12931_2018_945_MOESM1_ESM.tif]

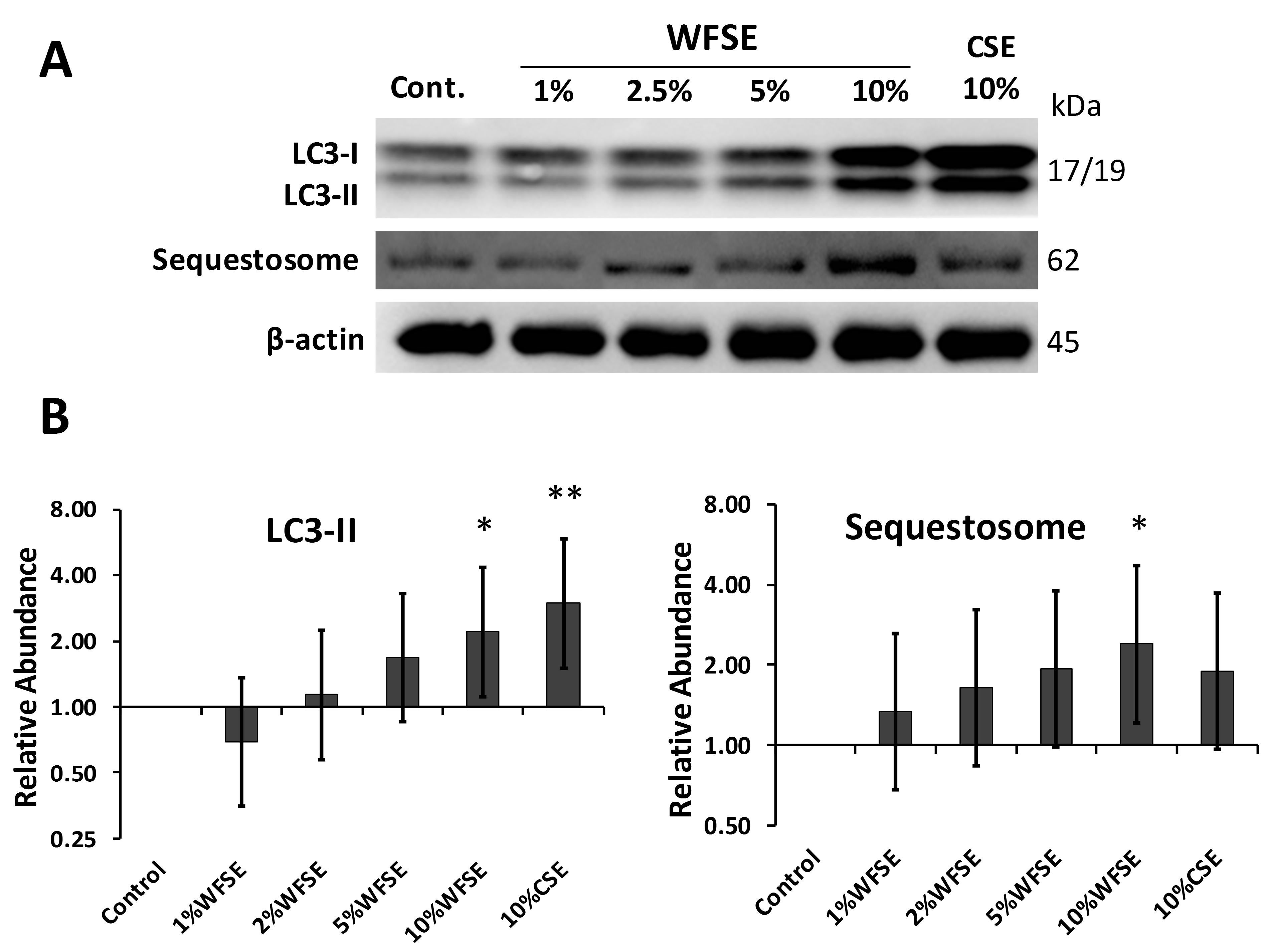

Supplement: Supplementary file 2 — Figure S2. THP1 macrophages demonstrate a block in autophagic flux when exposed to WFSE. A. THP1 macrophages (differentiated using 45 nM phorbol myristate acetate for three days) were exposed to wildfire smoke extract (WFSE) for 24 h and examined for modulation in autophagy via western blot analysis. As observed in the small airway epithelial cell model, the 10% WFSE exposure potentiates a block in autophagic flux in THP1 macrophages as evidenced by an increase in the essential autophagy protein Microtubule-Associated Protein 1A/1B-Light Chain-3-II (LC3-II; lower band), simultaneous with increased Sequestosome, which is normally degraded by the autolysosome. B. Histogram analyses of protein expression density scores. Protein expression was baselined to the abundance in the untreated sample, and normalized to the expression of β-actin. Intervals are 95% CI, and significance compared to the control sample can be identified when confidence intervals do not intersect 1 for the Y-axis. *, P ≤ 0.05; **, P ≤ 0.01 for n = 3 experiments. (TIF 633 kb) [file 12931_2018_945_MOESM2_ESM.tif]
