# Supplementary material for: Airway epithelial cells exposed to wildfire smoke extract exhibit dysregulated autophagy and barrier dysfunction consistent with COPD
Source: Respir Res. 2018 Nov 28;19:234. doi: 10.1186/s12931-018-0945-2 (PMC6263553; doi:10.1186/s12931-018-0945-2)
Supplement: Supplementary file 3 — Table S1. The main effects for bAEC barrier dysfunction elicited by WFSE.Differentiated primary bronchial airway epithelial cells were examined for barrier dysfunction by quantifying trans-epithelial electrical resistance (ionic conductance of the paracellular pathway) and permeability of fluorescent NaFl tracers (molecular flow of the paracellular pathway) applied across the epithelial layer in a transwell culture system. Results are presented for the individual exposures across the assay period intervals (i.e. incorporating each time interval from 0 to 24 h) to identify the main effects for each treatment (n = 3). Data for each treatment is presented relative to the control and 10% cigarette smoke extract (10% CSE) exposures. (DOCX 19 kb) [file 12931_2018_945_MOESM3_ESM.docx]

| **Trans-epithelial electrical resistance (Ω.cm^2^)** | | | | | | | | | | | |
| --- | --- | --- | --- | --- | --- | --- | --- | --- | --- | --- | --- |
|  | **Marginal Means** | | | **Compared to Control** | | | | **Compared to 10% CSE** | | | |
|  | **Mean** | **-95% CI** | **+95% CI** | **Contrast** | **-95% CI** | **+95% CI** | **P-val** | **Contrast** | **Lower CI** | **Upper CI** | **P-val** |
| Control | 844 | 814 | 873 | Reference |  |  |  | 213 | 171 | 254 | < 0.001 |
| 1.0%WF | 769 | 740 | 798 | -75 | -116 | -33 | 0.001 | 138 | 97 | 179 | < 0.001 |
| 2.5%WF | 690 | 661 | 719 | -154 | -195 | -112 | < 0.001 | 59 | 18 | 100 | 0.006 |
| 5.0%WF | 630 | 601 | 659 | -213 | -255 | -172 | < 0.001 | -1 | -42 | 41 | 0.97 |
| 10%WF | 556 | 527 | 586 | -287 | -329 | -246 | < 0.001 | -75 | -116 | -33 | 0.001 |
| 10%CSE | 631 | 602 | 660 | -213 | -254 | -171 | < 0.001 | Reference |  |  |  |
| **Paracellular molecular permeability (µg/mL tracer)** | | | | | | | | | | | |
|  | **Marginal Means** | | | **Compared to Control** | | | | **Compared to 10% CSE** | | | |
|  | **Mean** | **-95% CI** | **+95% CI** | **Contrast** | **-95% CI** | **+95% CI** | **P-val** | **Contrast** | **Lower CI** | **Upper CI** | **P-val** |
| Control | 34.1 | 18.3 | 50 | Reference |  |  |  | -142.9 | -165.4 | -120.5 | < 0.001 |
| 1.0%WF | 37.1 | 21.2 | 53 | 3 | -19.5 | 25.4 | 0.79 | -140 | -162.4 | -117.5 | < 0.001 |
| 2.5%WF | 38.3 | 22.4 | 54.2 | 4.2 | -18.3 | 26.6 | 0.71 | -138.8 | -161.2 | -116.3 | < 0.001 |
| 5.0%WF | 250.1 | 234.2 | 266 | 216 | 193.5 | 238.4 | < 0.001 | 73.1 | 50.6 | 95.5 | < 0.001 |
| 10%WF | 342.2 | 326.3 | 358.1 | 308.1 | 285.6 | 330.5 | < 0.001 | 165.2 | 142.7 | 187.6 | < 0.001 |
| 10%CSE | 177.1 | 161.2 | 192.9 | 142.9 | 120.5 | 165.4 | < 0.001 | Reference |  |  |  |
